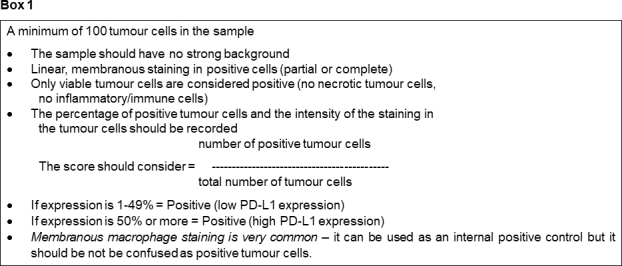# Critical Appraisal of Programmed Death Ligand 1 Reflex Diagnostic Testing: Current Standards and Future Opportunities

**DOI:** 10.1016/j.jtho.2018.09.025

**Published:** 2019-01

**Authors:** Matthew P. Humphries, Stephen McQuaid, Stephanie G. Craig, Victoria Bingham, Perry Maxwell, Manisha Maurya, Fiona McLean, James Sampson, Patricia Higgins, Christine Greene, Jacqueline James, Manuel Salto-Tellez

**Affiliations:** a Molecular Pathology Programme, Centre for Cancer Research and Cell Biology, Queen’s University, Belfast, Ireland, United Kingdom; b Cellular Pathology, Belfast Health and Social Care Trust, Belfast City Hospital, Belfast, Ireland, United Kingdom; c Northern Ireland Biobank, Centre for Cancer Research and Cell Biology, Queen’s University, Belfast, Ireland, United Kingdom

**Keywords:** Validation, Programmed death ligand 1, Clinical workflow, RNAscope, Image analysis

## Abstract

**Introduction:**

Patient suitability to anti–programmed death ligand 1 (PD-L1) immune checkpoint inhibition is key to the treatment of NSCLC. We present, applied to PD-L1 testing: a comprehensive cross-validation of two immunohistochemistry (IHC) clones; our descriptive experience in diagnostic reflex testing; the concordance of IHC to in situ RNA (RNA-ISH); and application of digital pathology.

**Methods:**

Eight hundred thirteen NSCLC tumor samples collected from 564 diagnostic samples were analyzed prospectively, and 249 diagnostic samples analyzed retrospectively in tissue microarray format. Validated methods for IHC and RNA-ISH were tested in tissue microarrays and full sections and the QuPath system were used for digital pathology analysis.

**Results:**

Antibody concordance of clones SP263 and 22C3 validation was 97% to 98% in squamous cell carcinoma and adenocarcinomas, respectively. Clinical NSCLC cases were reported as PD-L1–negative (48%), 1% to 49% (23%), and more than 50% (29%), with differences associated to tissue-type and *EGFR* status. Comparison of IHC and RNA-ISH was highly concordant in both subgroups. Comparison of digital assessment versus manual assessment was highly concordant. Discrepancies were mostly around the 1% clinical threshold. Challenging IHC interpretation included 1) calculating the total tumor cell denominator and the nature of PD-L1 expressing cell aggregates in cytology samples; 2) peritumoral expression of positive immune cells; 3) calculation of positive tumor percentages around clinical thresholds; and 4) relevance of the 100 malignant cell rule.

**Conclusions:**

Sample type and *EGFR* status dictate differences in the expected percentage of PD-L1 expression. Analysis of PD-L1 is challenging, and interpretative guidelines are discussed. PD-L1 evaluations by RNA-ISH and digital pathology appear reliable, particularly in adenocarcinomas.

## Introduction

Immune checkpoint blockade therapy is a new paradigm in cancer treatment with durable tumor regression and prolonged stabilization of disease in patients with advanced cancers, including NSCLC.^1^, ^2^ The binding of programmed death-1 receptor (PD-1) to its ligand programmed death ligand 1 (PD-L1) plays a pivotal role in the ability of tumor cells to evade the host's immune system.^3^ PD-L1 may be highly expressed on the surface of tumor cells. Binding of PD-1 with PD-L1 inhibits T cell activation, allowing immunosuppression and neoplastic growth.^4^ Blockade of this interaction has yielded long-lasting clinical benefits in many patients. Despite the clinical efficacy observed, many cancer patients do not respond to PD-1/PD-L1 checkpoint inhibition. Along with PD-L1 expression, other markers such as microsatellite instability and tumor mutation burden, as well as understanding the role of other markers and the function of tumor infiltrating immune and stromal cells in the tumor microenvironment may be important in predicting clinical benefit of immune checkpoint inhibition and determining optimal combination therapies.^5^, ^6^, ^7^, ^8^, ^9^, ^10^

In 2016, pembrolizumab was given European Medicines Agency approval for first-line treatment of metastatic or locally advanced squamous and non-squamous NSCLC.^11^ Patients with an identified PD-L1 expression may be treated with pembrolizumab based on PD-L1 expression equal to or greater than 1% in second-line treatment or where PD-L1 expression is equal to or greater than 50% in first-line treatment.^11^, ^12^

There are many PD-L1 antibodies and scoring systems currently in use.^13^ Four PD-L1 assays with four distinct antibodies and two separate automated staining patterns are registered with the U.S. Food and Drug Administration. This raised many questions about inter- and intralaboratory variations in assessment of PD-L1 expression. The current literature however, appears to have reached a consensus.^14^, ^15^, ^16^ Dako 28-8, Dako 22C3, and Ventana SP263 are closely similar, showing strong and comparable expression on the tumor cells of the NSCLC, but delineating fewer immune cancer-associated cells, and thus have higher sensitivity to the performance of Ventana SP142.^17^

Despite this apparent similarity, the validation and application of PD-L1 immunohistochemistry (IHC) for diagnostics is fraught with challenges. Subtle discordance between antibodies, separation of tumor cell versus inflammatory cell PD-L1 chromogenic signals, apparent focal signal mislocalization from the membrane to the cytoplasm, scoring of percentages of expression (particularly around the thresholds of clinical relevance less than 1% and 50%) or specific dilemmas associated to small biopsy and cytology samples.

Hereby we present a comprehensive assessment of the PD-L1 IHC diagnostic test in NSCLC at different levels: (1) a comparative cross-validation of the two more widely used antibodies, namely the Dako 22C3 and Ventana SP263 clones; (2) a description of clinical PD-L1 reflex testing in the first 564 cases in an accredited laboratory; (3) the concordance of PD-L1 overexpression by IHC versus PD-L1 upregulation by in situ RNA (RNA-ISH); and (4) the potential role of digital pathology in the automated scoring using open source available software (QuPath).

## Materials and Methods

### Comparative Validation

#### Clinical Samples

The total number of cases submitted for PD-L1 assessment as a reflex test was 583 over a 12 month period, across four National Health Service trusts in Northern Ireland. Five hundred sixty-four patient samples had adequate or complete data for retrospective analysis. The sample types included formalin-fixed paraffin-embedded cell blocks (n = 88), bronchoscopic and core biopsy specimens (n = 429), and surgical resections (n = 66). The reflex testing (in adenocarcinoma) included PD-L1 protein overexpression, ALK receptor tyrosine kinase (ALK) IHC, and *EGFR* mutational status.

#### Research Samples

A total of 249 cancer samples, represented by 120 tissue cores of lung adenocarcinoma and 114 cores of lung squamous cell carcinomas in a tissue microarray (TMA) format as well as 15 whole-face sections from patients who underwent surgery with curative intent from 2005 to 2015 at The Belfast Health and Social Care Trust were used.

Ethical approval was obtained and tissue was acquired through the Northern Ireland Biobank (reference: 12-00168). For adenocarcinoma, predominant histologic pattern (solid, lepidic, acinar, papillary, and micropapillary) was determined according to the 2015 WHO classification.^18^ For squamous cell carcinoma grading, we used well, moderate, and poorly differentiated categories. The TMA blocks were prepared using 1.0-mm tissue cores as described previously and using national guidelines.^19^, ^20^ *EGFR* mutation data obtained using COBAS or Sanger sequencing was available in 250 cases of adenocarcinoma. ALK fusion protein expression data was obtained using ALK IHC, only in adenocarcinoma, with the D5F3 clone on a Ventana BenchMark platform and was positive in 7 of 407 adenocarcinoma cases. This was complemented by a cohort of 15 whole-face sections (8 adenocarcinomas and 7 squamous cell carcinomas).

#### IHC Staining

Three-micrometer-thick sequential histologic tumor sections were obtained from representative formalin-fixed paraffin-embedded tumor blocks (whole-face or TMA) and used for IHC analysis. IHC was performed using an automated staining system (Ventana BenchMark) with antibodies against PD-L1 (SP263 clone, Ventana, CC1 pre-treatment for 64 minutes, Ventana Optiview detection protocol) or using a Dako automated platform with antibody to the 22C3 clone of PD-L1. Both systems used a diaminobenzidine reaction to detect antibody labeling and hematoxylin counterstaining.

#### Strategy of Comparative Validation

Serial sections from lung adenocarcinoma or lung squamous carcinomas (whole-face or TMAs) were stained for PD-L1 (SP263 clone) in The Northern Ireland Molecular Pathology Laboratory (Belfast) or for PD-L1 (22C3 clone) in Southampton (University Hospital of Southampton, NHS Trust). Assessment of PD-L1 was performed by two individuals (M.S.T. and S.M.) who have received training and are certified competent for PD-L1 scoring in accordance with recognized parameters. In each whole-face section or TMA core the criteria in box 1 (Supplemental Table 1) were used in the scoring assessments.^21^ Internal positive control tissues were to represent the different expression patterns of PD-L1 as well as tonsil tissue with strong expression observed in crypts and weaker expression in follicles.

### PD-L1 Testing in Routine Practice

From April 2017 to March 2018, 564 patient samples were tested and reports issued. All samples were clinically assessed by teams of two individuals who received training and are certified competent for PD-L1 scoring. Sections from a small internal TMA consisting of four cores (representing PD-L1 expression levels of more than 50%, 1% to 49%, and less than1%, as well as tonsil) were used in each test run to assess specificity and sensitivity and intra-run reproducibility.

### RNA-ISH Assay

#### Method

Automated RNAscope for PD-L1 was performed on sections from the adenocarcinoma and squamous cell carcinoma TMAs on a Leica Bond RX platform. Briefly, sections were cut at 4 μm, air dried overnight, baked at 60°C for 1 hour, dewaxed, and air-dried before pretreatments. For all tissue sections, a standard pretreatment protocol was used. Three RNAScope probes from Advanced Cell Diagnostics (ACD; Hayward, California) were used in this study: positive-control probe Hs-PPIB (313908 Accession # NM_000942.4-4 – 139 - 989); and probe to the immune pathway–associated biomarker PD-L1 – Hs-CD274 (600868 Accession # NM_014143.3 – sequence region 124 - 1122) were also used to stain the lung TMAs. Also a negative-control probe DapB (312038 Accession # NM_EF191515) was tested in a subset of adenocarcinoma and squamous cell carcinoma TMA cases with no expression observed on any cores. Detection of specific probe binding sites done was with the RNAScope 2.5 HD Reagent kit-brown from ACD (Cat. No. 322300).

#### Materials and Scoring System

Thirty lung adenocarcinomas and 30 lung squamous cell carcinomas in TMA format were assessed for overall expression of PD-L1 by IHC and by RNAscope. These cases in TMA format were selected according to immediate availability, thus, they represent a cross-section of all samples submitted to a routine diagnostic lab. For IHC, cases were classified as less than 1%, 1% to 49%, or more than 50% PD-L1 expression on tumor cells, taking into account all available tumor in all cores for the individual cases. For PD-L1, mRNA expression by RNAscope cases were classified as less than 1% expression on tumor cells (no staining or just one dot per call in a few tumor cells), 1% to 49% of tumor cells have two or more dots per cell, or more than 50% of tumor cells have two or more dots per cell with many foci having abundant dots-per-cell. All RNAscope assessments were performed by two individuals (S.M. and V.B.) who are experienced users of the RNAscope assay.^22^, ^23^

### Image Analysis of PD-L1 Expression

Digital image analysis of the IHC stained TMA slides or clinical cases was performed using an in-house image analysis program called QuPath.^24^, ^25^ All slides were scanned on an Aperio AT2 digital scanner at ×40 and imported into the program. Following an established method, tissue detection was performed by identifying the tissue for cellular analysis.^24^ Samples that contained less than 100 tumor cells were excluded from analysis. Rigorous quality control (QC) steps were taken to remove necrosis or keratin, tissue folds, and entrapped normal structures; this was confirmed by a second reviewer with frequent consultation. Carbon pigment was also edited out to avoid these deposits being interpreted as positive cells. Furthermore, PD-L1 expression by macrophages is well recognized and these were manually edited out where possible to minimize the risk of false-positive detections. For manual evaluation, a positive cell was defined as one which showed a pattern of membrane staining of any intensity. QuPath’s ability to classify cell types within each tissue core was applied to distinguish tumor and stroma compartments.

Quantitation was conducted as previously described.^24^ Cores included in QuPath analysis were also manually assessed in a blinded fashion as described above and a percentage of tumor-positive cells recorded.

## Results

### Comparative Validation of PD-L1

**Figure 1:**
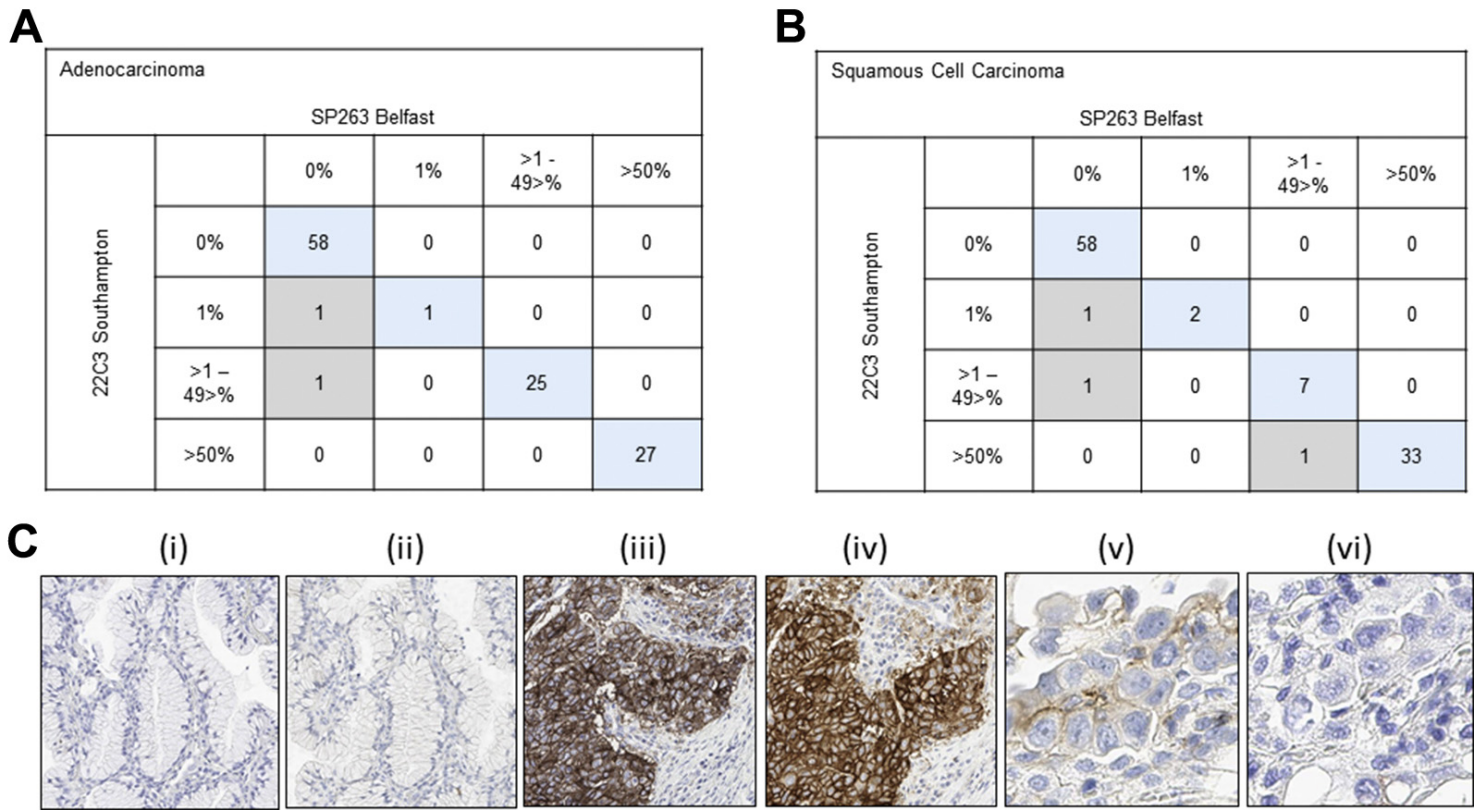
Summary of the validation on tissue microarrays of the programmed death ligand 1 (PD-L1) SP263 clone on Ventana BenchMark against the Dako PD-L1 22C3 clone on the Dako Autostainer Link 48 in (*A*) lung adenocarcinomas and (*B*) squamous cell carcinomas. A very high level of concordance was observed. For adenocarcinomas, there were 111 of 113 tissue microarray (TMA) cores (98%); and for squamous cell carcinomas, there were 100 of 103 TMA cores (97%). (*C*) For (i) 22C3 and (ii) SP263 there is no PD-L1 expression in lung adenocarcinoma, whereas (iii) 22C3 and (iv) SP263 show comparable expression of more than 50% on tumor epithelium in a squamous cell carcinoma. And in (v) 22C3, two or three tumor epithelial cells are expressing PD-L1, but this expression was not observed in a serial core with the SP263 clone (vi).

The concordance between the assessment of PD-L1 expression (SP263 clone) on a Ventana BenchMark platform and PD-L1 expression (22C3 clone) on a Dako Autostainer platform is shown in Figure 1. Of TMA cores that were available for assessment, 111 of 113 TMA cores from adenocarcinomas were fully concordant with an overall concordance rate of 98% (Fig. 1*A*). One hundred of 103 TMA cores from squamous cell carcinomas were fully concordant with an overall concordance rate of 97% (Fig. 1*B*). In 58 adenocarcinomas and 58 squamous cell carcinomas, no expression was seen with either clone (Fig. 1*C*, parts i and ii). In 27 adenocarcinoma and 33 squamous cell carcinomas, concordance was observed at over 50% expression (Fig. 1*C*, parts iii and iv). In two cases of adenocarcinoma, the 22C3 clone showed expression on a few tumor epithelial cells, which was assessed in the cores as 1% and 2%, respectively (Fig. 1*C*, part v). The same cell populations did not show expression with the SP263 clone (Fig. 1*C* part vi). Similarly, two squamous cell carcinoma TMA cores showed very low level expression with the 22C3 clone which were not observed with the SP263 clone. In a single squamous cell carcinoma TMA core, the 22C3 clone showed more than 50% PD-L1 expression (65%); however, only 1% to 49% expression (35%) PD-L1 expression was observed with the SP263 clone.

For both antibody clones, overall scores for PD-L1 expression were fully concordant for 15 of 15 whole-face resections of either adenocarcinoma or squamous cell carcinoma of the lung. Five cases were assessed as less than 1%, six cases as 1% to 49%, and four cases as more than 50%.

### PD-L1 Testing in Routine Practice

**Figure 2:**
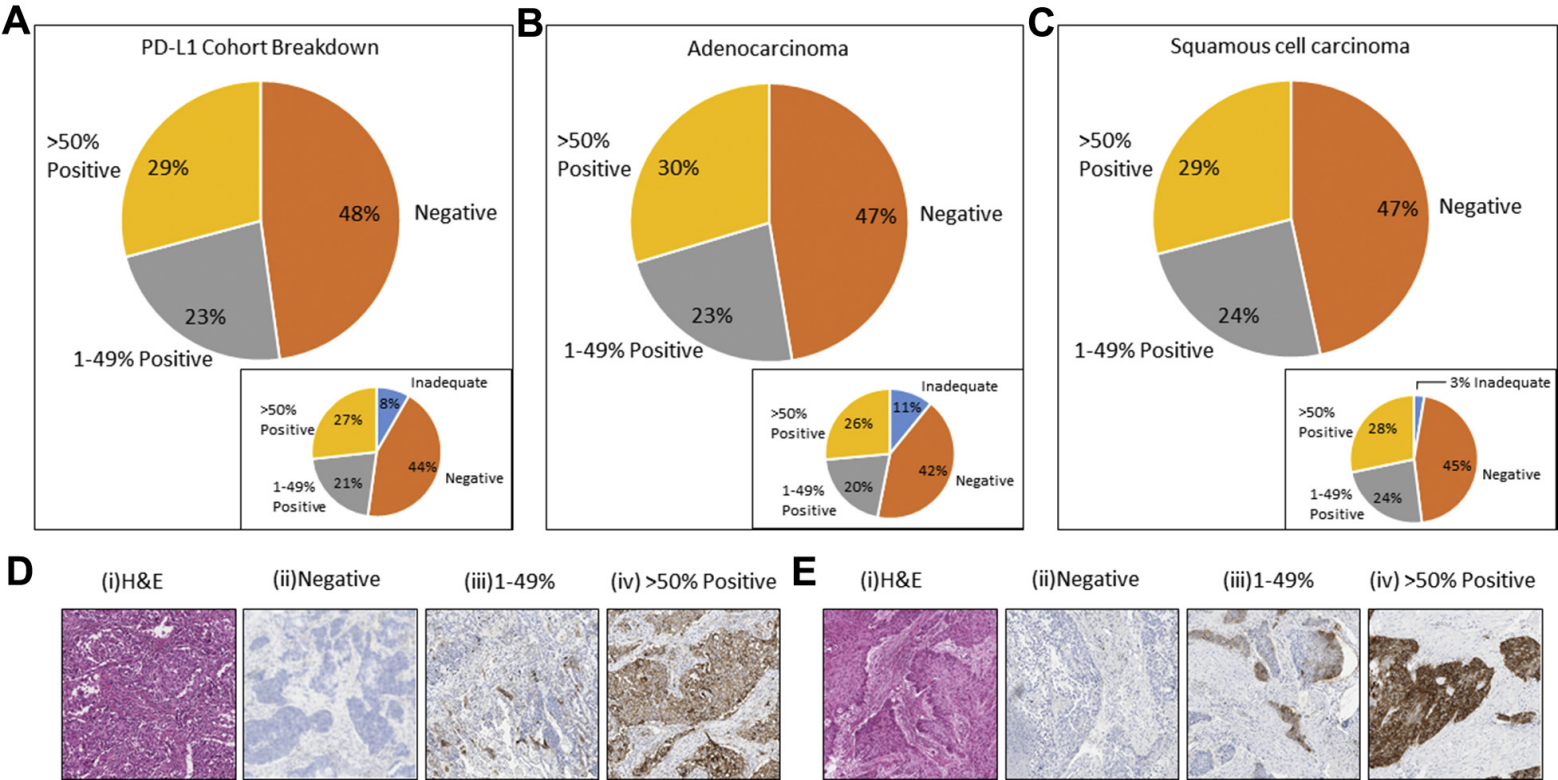
Comparable categorical distribution of programmed death ligand 1 (PD-L1) expression. (*A*) Within all 564 clinical cases. (*B*) In adenocarcinomas. (*C*) In squamous cell carcinomas. Inadequate cases were defined as those which had insufficient tumor content (<100 malignant cells) available for analysis. (D, E) Left-to-right display representative images of (i) hematoxylin and eosin staining (H&E), (ii) negative PD-L1 expression, (iii) 1% to 49% PD-L1 expression, and (iv) more than 50% positive PD-L1 expression for adenocarcinoma and squamous cells carcinoma, respectively.

Five hundred sixty-four of 583 samples were available for analysis. Nineteen cases were found to be inadequate for assessment (less than 100 tumor cells in the sample) (Fig. 2*A*, inset). Eleven percent of the adenocarcinomas were inadequate for PD-L1 testing, with 3% determined inadequate for squamous cell carcinoma histology (Figs. 2*B* and *C* insets, respectively).

Of the 564 cases tested, 48% were PD-L1–negative (less than 1% positive). With categories 1% to 49% and more than 50%, PD-L1 positivity was reported as 23% and 29%, respectively (Fig. 2*A*). There was little difference in PD-L1 categorization of cases within the two main histologic subtypes, adenocarcinoma and squamous cell carcinoma (Figs. 2*B* and *C*). Representative hematoxylin and eosin staining as well as images of PD-L1 categories are shown in Figures 2*D* and *E* for adenocarcinomas and squamous cell carcinomas, respectively.

**Figure 3:**
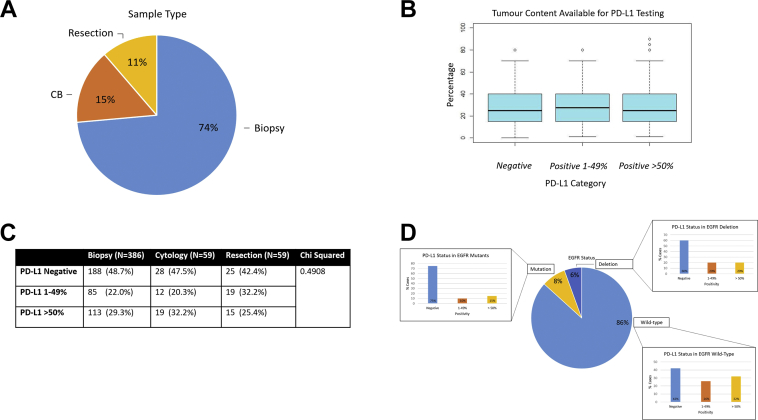
Range of sample types tested, including tumor content available for programmed death ligand 1 (PD-L1) testing. (*A*) The range of samples types received for testing, resection, biopsy and cell block (CB). (*B*) Confirms that tumor content availability did not significantly affect the PD-L1 category determined. (*C*) PD-L1 expression according to sample type. *p* value is determined by the chi-square test. (*D*) PD-L1 expression within the cases of differing EGFR status.

Five hundred sixty-four cases included 74% biopsies, 15% cytology cell blocks, and 11% surgical resections (Fig. 3*A*). The positivity of PD-L1 expression differed across sample types. Within the more than 50% positivity clinical category there was relative consistency in percentages of expressing cases (29% biopsy cases, 32% cytology cases, and 25% surgical resections). In the 1% to 49% clinical category, the percentage expressing PD-L1 is diminished in biopsies (22%) and cytologies (20%) when compared with resections (33%), as shown in Figure 3*C*.

Of the cases with PD-L1 categories determined, *EGFR* mutational status was also investigated. Eighty-six percent of the cases were found to be wild-type for *EGFR*. Eight percent of cases were determined to have point mutations, with 6% *EGFR* deletions. The PD-L1 expression within the *EGFR* wild-type, mutation or deletion was also evaluated (Fig. 3*D*).

The PD-L1 expression category was not influenced by the availability of tumor content if more than 100 cells were available. Figure 3*B* shows the range of tumor cells available for analysis and the clinical PD-L1 category reported. There was no significant difference across the PD-L1 clinical categories relative to tumor content available for testing.

### Challenges in Routine PD-L1 Assessment

There are several challenges to routine PD-L1 assessment:

1. Percentage of positive cells in cytology samples — the need for a “background stain.” PD-L1 expression is reported as a percentage of existing malignant cells that are expressing the antibody. Although the denominator of this equation (total number of malignant cells) is clear in histology samples, in cytology specimens this may not be so obvious, as shown by a malignant lung epithelial IHC stain thyroid transcription factor 1 (Fig. 4*A*).

**Figure 4:**
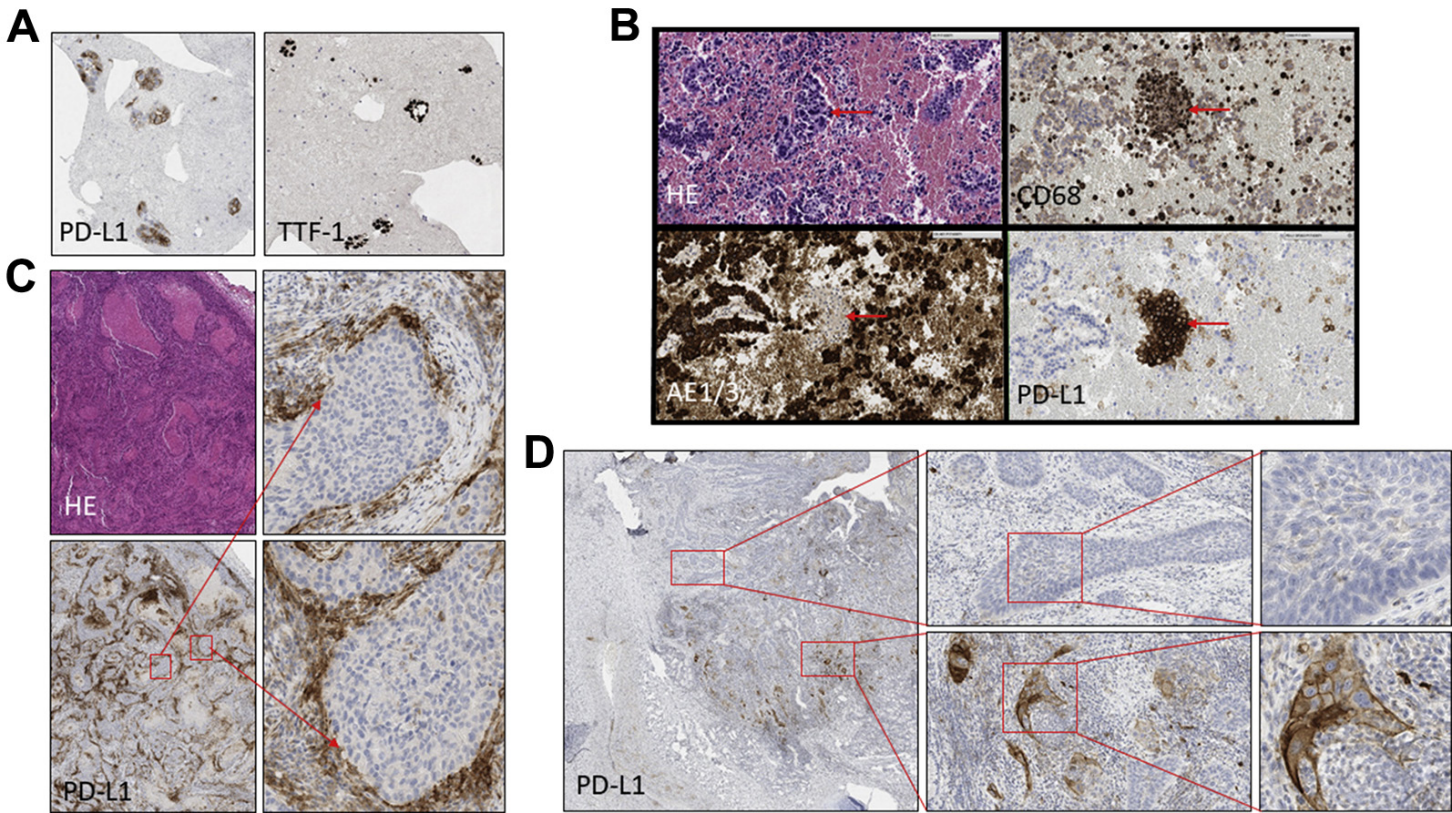
Challenges with programmed death ligand 1 (PD-L1) assessments. (*A*) PD-L1 expressing tumor cells in a cell block which are confirmed by TTF1 expression a highly specific marker for primary lung adenocarcinomas. The case was reported as more than 50% PD-L1 expression in tumor cells. (*B*) PD-L1 expression in foci of cells in a lung cell block which, when phenotyped with macrophage and epithelial markers, were identified as macrophages. The case was reported as PD-L1–negative. (*C*) Resection of adenocarcinoma in which there is strong expression of PD-L1 on lymphocytes which “hug” the tumor. (*D*) Resection sample from squamous cell carcinoma shows distinct heterogeneity of PD-L1 expression ranging from absent to more than 50% expression in various fields across the tumor bed. TTF-1, thyroid transcription factor 1; HE, hematoxylin and eosin.
2. Nests of positive cells on cytology — macrophages or malignant cells. Figure 4*B* shows a case in which the nature of the positive PD-L1 cells had to be elucidated by a comparison of HE, CD68, AE1/3, and PD-L1 in consecutive sections of a cytology cell block.
3. Tumor versus inflammation — the “hugging effect.” Similarly, sometimes the presence of PD-L1 strongly positive inflammatory cells around nests of malignant epithelial cells is prominent (Fig. 4*C*). Here, the rule is not to call something epithelial-positive unless there is unequivocal evidence that the staining is also present in the malignant cells.
4. Calculation of percentages around the “clinical thresholds.” Unlike the 2+ category when scoring of Her2 in breast or gastric cancer, PD-L1 does not have any “threshold/buffer” score on which we can refer to other methods and, as such, there is a degree of uncertainty when a sample has “a few positive cells” (threshold of less than 1% to 2.7%, an occurrence happening in 9 of 564 of the cases in our reported experience), or “approximately half of the malignant cells are positive” (threshold 40% to 60%).
5. One hundred malignant cell minimum — when is this applicable? This rule aims to achieve a “minimum representation” of the sample, particularly in view of the notorious heterogeneity of PD-L1 expression identified in some resection specimens (Fig. 4*D*). In cases of less than a 100-cell minimum in which the cytology or biopsy sample is the only one available, the percentage of positive malignant cells should be reported with the diagnostic label of “unsatisfactory.” These are summarized in Supplementary Table 1.

### Potential Use of Image Analysis and RNA-ISH for PD-L1 Expression

**Supplemental Figure 1:**
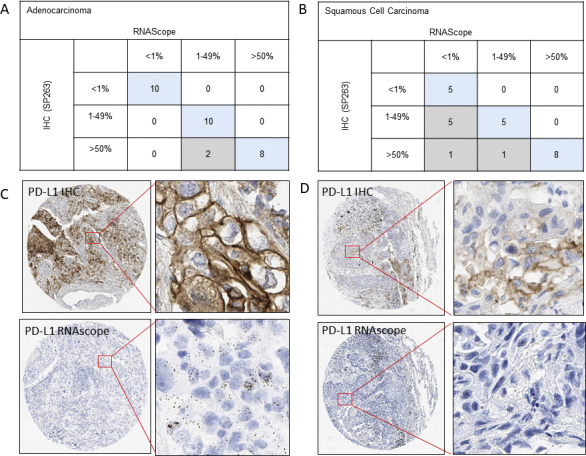


The concordance of PD-L1 expression by IHC (SP263 clone) and RNA-ISH in TMAs is shown in Supplementary Figure 1. Excellent concordance was seen between ICH and RNA-ISH. One case which by IHC showed more than 50% expression did not show any mRNA expression by RNA-ISH.

**Supplemental Figure 2:**
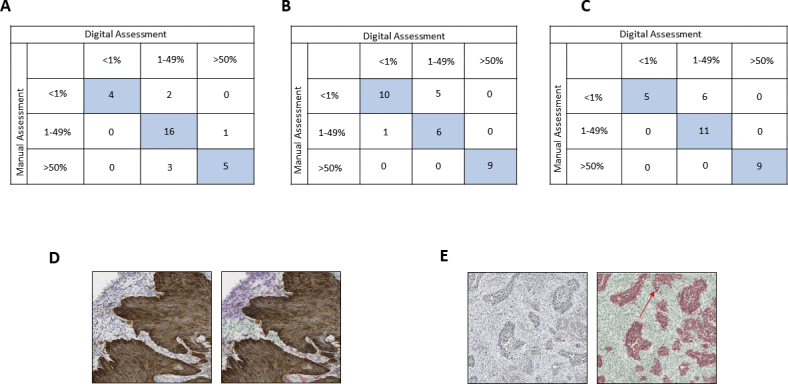


The concordance of manual PD-L1 assessment (the current gold standard) with that of digital pathology (QuPath) is shown in Supplementary Figure 2. In addition to the cases from routine diagnostics, we assessed 62 cases across all three clinical categories in our squamous cell carcinoma and adenocarcinoma TMAs. Supplementary Figure 2*D* and *E* show concordant and discordant cases between manual and digital assessment in a case which had high PD-L1 expression and Low PD-L1 expression, respectively.

## Discussion

Despite the equivalence between PD-L1 antibodies, the reported expression rates in lung adenocarcinoma vary greatly.^26^, ^27^, ^28^, ^29^ This may be a reflection of (1) the sample cohorts analyzed; (2) the proportion of different clinical sample-types in the studies; and (3) the intrinsic difficulty of this test, particularly in the areas of “diagnostic threshold.” Our clinical validation confirms the equivalence of the 22C3 and SP263 PD-L1 antibody clones, perhaps highlighting that when other variables are corrected for (same interpretative team, same rules of interpretation, and same samples) the reported antibody equivalence should be obvious. In our opinion, this calls for the development of “reference materials” for multicenter validation of tests such as PD-L1 IHC.

The clinical experience on our first 564 cases indicates that, if the validation is correct, the sample type may influence the result. However, the histologic subtype does not have any relevance in the validation or in the final numbers. The PD-L1 expression pattern seen in resections does not mirror exactly that seen in the cytology and biopsy samples. This may be explained by the heterogeneity of PD-L1 expression combined with an increased tissue area for assessment which leads to an increased reporting of 1% to 49% cases from resection specimens, closer to the percentages in clinical trials. The PD-L1 negative rate of 48% we report here is indifferent to that reported in clinical trials (e.g., KEYNOTE-010 and KEYNOTE-024). This can be explained by the fact that in trials there is an attempt to retrieve a new sample, thus increasing the availability of tissue not tested for other purposes. Furthermore, there is a lack of acceptance of fine needle aspirates for testing in clinical trials.^11^ Our real-world samples are different, with a predominance of small volumes already tested for other biomarkers, which possibly brings down the numbers of cases in the 1% to 49% category.

Our routine experience highlights potential areas of diagnostic difficulty. We suggest the following possible solutions, which can be consolidated into 3 points: (1) Use other IHC markers when needed for interpretative support. As a result, our policy for cytology samples is to request, when possible, a “malignant lung epithelial IHC stain” (TTF-1 or similar) to establish the true cellular background and carry out the most accurate calculation. Occasionally, upon encountering cases with a “hugging effect”, the use of epithelial/inflammatory IHC antibodies can be justified, and also evokes the potential use of “double staining.” (2) Consult on the sample with a trained colleague in quantitation when close to clinical thresholds. And (3), make judicious use of the 100-cell threshold rule in the patients’ clinical context. In cases in which the cytology or biopsy sample is the only one available, we report the percentage of positive malignant cells if we detect a degree of expression to avoid the label of “unsatisfactory,” as this may be of clinical relevance. This is particularly important in cases where no other specimen is available and/or further sampling may not be possible.

In difficult cases where positive immune cells surround tumor nests, a so-called “hugging effect” is evident; there may be a requirement for the application of other IHC staining to confirm epithelial cell positivity. The advantage of this is primarily a confident reporting of PD-L1 expression for the patient, with the main disadvantages being the time and cost of IHC double staining and/or multiplexing in routine diagnostics. In cases where there is a difficulty in deciding if the PD-L1 signal originates in the malignant epithelial cells or in the surrounding immune cells, we suggest the sample is regarded as positive only if there is “unequivocal evidence” of epithelial expression.

The concordance between PD-L1 IHC and RNA-ISH is very good. It would be interesting to see if the small percentage of discrepancy actually confers higher predictive value to the RNA-based analysis in treated sample collections. RNA-ISH can be a quantitative methodology and, as such, may represent a way forward to bring consistency and concordance to this test; thus making it a realistic diagnostic option.

The subjective assessment of PD-L1 positivity may provide an arena in which use of image analysis as a companion diagnostic aid could be beneficial. The accuracy and reproducibility of digital pathology may be considered overall or in difficult cases. In our hands, digital pathology is demonstrably accurate in the analysis of PD-L1. The gold standard of assessment, namely the human diagnostician, is able to qualify the discrepancy upon review of the tumor classifier applied by digital pathology. The application of robust tumor classifiers and deep learning in digital pathology may solve the disparity between pathologist and machine or, when applied to clinical trial material, show the superiority of the in silico approach to scoring, as other studies are beginning to suggest.^30^ In any case, the application of digital pathology in clear-cut cases, thus requiring little pathologist input, would enable pathologist to spend more time analyzing cases within the difficult diagnostic thresholds.

One of the main characteristics of PD-L1 IHC scoring is the common denominator, that is, the total number of malignant cells. This can be calculable with relative ease in histology samples, but it is significantly more challenging in cytology samples with discohesive malignant cells in benign, reactive cellular backgrounds. In this context, the cross-reference to cancer cell–specific IHC (such as TTF-1) is very important and unfortunately not always available when the test is performed in referral laboratories.

In summary, hereby we present a successful cross-validation of PD-L1 as a diagnostic test, we discuss the areas of interpretative difficulty associated to biopsies and cytology, and we explore the pathway to make the test more objective and quantifiable by incorporating RNA-ISH and digital pathology scoring interpretation. This may represent a validation footprint for other future examples on therapeutic IHC in solid tumors.

## Figures and Tables

**Supplemental Figure 3:**