# Supplementary material for: Critical Appraisal of Programmed Death Ligand 1 Reflex Diagnostic Testing: Current Standards and Future Opportunities
Source: J Thorac Oncol. 2019 Jan;14(1):45–53. doi: 10.1016/j.jtho.2018.09.025 (PMC6328626; doi:10.1016/j.jtho.2018.09.025)
Supplement: Supplementary Table 1 [file mmc1.docx]

| **Diagnostic Dilemma** | **Potential solution** |
| --- | --- |
| How many malignant cells are there in a cytology sample | Evaluate PD-L1 IHC in comparison with a malignant lung epithelial IHC stain |
| Nests of positive cells on cytology – macrophages or malignant cells | Evaluate PD-L1 IHC in comparison with other IHC stains |
| Tumour vs inflammation – the “hugging effect”. | Evaluate the PD-L1 IHC against consecutive epithelial/immune IHC stains, or potentially a double IHC stain. |
| Calculation of percentages around the “clinical thresholds”. | Consult with a second reporting pathologist |
| 100 malignant cell minimum | If there is positivity in the malignant cells, report the percentage even in samples with less than 100 cells |

Table ST1 – Routine diagnostic dilemmas and potential solutions with PD-L1 IHC
